# Lack of efficacy of fenbendazole against *Giardia duodenalis* in a naturally infected population of dogs in France

**DOI:** 10.1051/parasite/2022048

**Published:** 2022-10-28

**Authors:** Hugo Kaufmann, Lionel Zenner, Slimania Benabed, Marie-Thérèse Poirel, Gilles Bourgoin

**Affiliations:** 1 Université de Lyon, VetAgro Sup – Campus Vétérinaire de Lyon, Laboratoire de parasitologie vétérinaire 1 avenue Bourgelat, BP 83 F-69280 Marcy l’Etoile France; 2 Université de Lyon, Université Lyon 1, CNRS, UMR 5558, Laboratoire de Biométrie et Biologie Évolutive F-69622 Villeurbanne France

**Keywords:** Giardiosis, Fenbendazole, Dogs, Therapeutic efficacy, Zoonosis

## Abstract

Giardiosis is a worldwide intestinal parasitosis, affecting both humans and animals. Treatment in dogs remains limited and the lack of efficacy of the few approved medications is a rising concern. In this study, 23 dogs raised by veterinary students and naturally infected with *Giardia duodenalis* were treated in home conditions with fenbendazole (50 mg/kg orally for 5 consecutive days). Fecal samples were collected immediately before treatment (FS1), 2–4 days after treatment (FS2) and 8–10 days after treatment (FS3). *Giardia duodenalis* cyst excretion was measured quantitatively by direct immunofluorescence assay (DFA) at FS1, FS2 and FS3. Molecular typing with a nested PCR targeting the SSU _r_DNA locus was also performed at FS1 and FS2. Fecal consistency improved in 16/21 dogs (76%) and mean cyst shedding was reduced by 84% after treatment. However, only 8/23 dogs (35%) achieved therapeutic success (≥90% reduction of cysts) and only 4/23 dogs (17%) had complete elimination of *G. duodenalis*. Molecular typing showed that dogs harbored only canine-specific assemblages, with a high prevalence of assemblage C in analyzed samples (30/39). We also detected different assemblages after treatment and nucleotide substitutions in assemblage C sequences that have not been described previously. Eight to ten days after treatment, high *Giardia* cyst excretion was measured, suggesting possible reinfection despite hygiene measures and/or multiplication. These data suggest that fenbendazole treatment may improve fecal consistency but has limited therapeutic efficacy against giardiosis in this population of dogs. Further research is still needed to assess the efficacy of fenbendazole against canine giardiosis.

## Abstract (fr)

La giardiose est une parasitose intestinale mondiale, touchant à la fois l’homme et les animaux. Chez le chien, le traitement reste limité et le manque d’efficacité des quelques médicaments autorisés inquiète de plus en plus. Dans cette étude, 23 chiens d’étudiants vétérinaires et infectés naturellement par *Giardia duodenalis* ont été traités en conditions réelles avec du fenbendazole (50 mg/kg par voie orale pendant 5 jours consécutifs). Des échantillons de selles ont été collectés juste avant le traitement (FS1), 2–4 jours après traitement (FS2) et 8–10 jours après traitement (FS3). L’excrétion de kystes de *G. duodenalis* a été mesurée quantitativement par immunofluorescence directe (IFD) à FS1, FS2 et FS3. Un génotypage par PCR nichée ciblant le locus SSU ADN_r_ a également été réalisé à FS1 et FS2. La consistance des selles a été améliorée chez 16/21 (76 %) chiens et la moyenne d’excrétion des kystes a été réduite de 84 % juste après le traitement. Seulement 8/23 (35 %) chiens ont atteint un succès thérapeutique (≥ 90 % de réduction d’excrétion de kystes) et 4/23 (17 %) chiens ont eu une élimination complète de *G. duodenalis*. L’analyse des séquences a montré que les chiens présentaient seulement des assemblages génotypiques spécifiques de l’espèce canine, avec une forte prévalence de l’assemblage C dans les échantillons analysés (30/39). Des changements d’assemblage après traitement et des substitutions nucléotidiques jamais décrites au sein de l’assemblage C ont également été observés. Huit à dix jours après traitement, une forte excrétion de kystes de *G. duodenalis* a été mesurée : malgré les mesures hygiéniques, une réinfection et/ou une multiplication semblent probables. Ces données suggèrent que le traitement au fenbendazole peut améliorer la consistance des selles mais a une efficacité thérapeutique limitée contre la giardiose dans cette population de chiens. Des recherches supplémentaires sont encore nécessaires pour évaluer l’efficacité du fenbendazole contre la giardiose canine.

## Introduction

*Giardia duodenalis* is an endemic intestinal parasite affecting more than 40 different animal species [37]. The prevalence of giardiosis in dogs varies in different studies according to the diagnostic method and location, but a meta-analysis conducted in 2014 showed a mean prevalence of 15.2% around the world [8]*. Giardia* likely remains one of the most common parasites in dogs. The highest prevalence is observed in young dogs, those living in a kennel environment, immunocompromized individuals and dogs with polyparasitism or concurrent infection [8, 40]. Diagnosis of giardiosis is challenging and may be achieved by direct fecal examination (fecal flotation or direct immunofluorescence assay) or by antigenic or PCR methods [44]. Currently, eight genotypes or assemblages of *G. duodenalis* are described (A to H) but only assemblages C and D are canine-specific [21]. Assemblages A and B present a zoonotic risk, although it is unlikely that a new human infection is due to transmission from dogs to humans. Indeed, studies show that dogs, especially shelter dogs, are rarely infected with zoonotic assemblages but rather with assemblages C and D [5, 6]. Many dogs have subclinical *G. duodenalis* infection, but giardiosis can lead to acute or chronic diarrhea and weight loss with delayed growth in puppies [24]. In such cases, treatment of giardiosis in dogs, based on both therapeutic drugs and hygiene measures to avoid recontamination, is recommended.

Metronidazole is the only drug approved in France for the treatment of giardiosis in dogs (50 mg/kg/d for 5–7 days) [2, 16]. However, few field efficacy trials are published [11, 12, 17, 18, 29] and *Giardia*-negative rates in dogs after metronidazole treatment are between 14.3% and 100%. The main limitation for veterinarians to the use of metronidazole in daily practice remains the adverse effects, including neurologic disorders (vestibulocerebellar ataxia) and teratogenicity. Neurotoxicity can appear when administering metronidazole at doses up to 40 mg/kg/d for a duration up to 3 days, which is a lower dose than recommended for the treatment of giardiosis [43].

Benzimidazoles can also be effective in eliminating *Giardia* infection in dogs and are commonly used for this indication. Febantel (15 mg/kg/d for 3–5 days) has an efficacy rate in dogs of between 33% and 100% [4, 12, 28]. Oxfendazole (11.3 mg/kg/d for 3 days) and albendazole (25 mg/kg/d for 7 days) also seem to effectively cure giardiosis. However, albendazole should be avoided because of its poor safety profile [12, 29, 46]. Fenbendazole, the active metabolite of febantel, remains the most commonly used therapy to cure *Giardia* infection in veterinary medicine because of its low cost, safety and efficacy. One of the most common fenbendazole-containing products, Panacur^®^ (MSD Animal Health, USA), is registered in most European countries to prevent and control *Giardia* infection in dogs [16], but it does not have that indication in France. The recommended dosage is 50 mg/kg bodyweight orally once daily for 3–5 days [10, 16]. *Giardia*-negative rates in dogs after fenbendazole treatment remain variable, ranging from 0% to 100% according to the study design and diagnostic method used [3, 11, 12, 18, 29, 38, 47].

Both nitroimidazoles and benzimidazoles have been used for years to manage giardiosis in dogs and also in humans. However, recent studies in human medicine challenge their efficacy as chemoresistance is thought to occur with extensive use. In a survey by the Hospital for Tropical Diseases in London, nitroimidazole-refractory disease rose from 15.1% in 2008 to 40.2% in 2013 [30]. In veterinary practice, few field efficacy trials in home conditions have assessed the efficacy of either metronidazole or fenbendazole to cure *Giardia* infection in dogs. Furthermore, despite the use of drugs and environmental hygiene measures, control of giardiosis in dogs is often challenging for veterinarians and owners, and treatment failure is not uncommon.

The purpose of the present study was to evaluate the efficacy of fenbendazole treatment in reducing *Giardia* cyst excretion and clinical signs in dogs naturally infected with *Giardia* at a veterinary college campus, where active circulation of *G. duodenalis* and high selective pressure are thought to occur. Genotyping of isolates was undertaken, not only to obtain information about the circulation of zoonotic species on the college campus, but also to look for a potential correlation between fenbendazole-refractory treatment and *Giardia* assemblage.

## Material and methods

### Ethics statement

All dogs were client-owned animals and all owners provided written consent. This study was conducted in compliance with the WAAVP guidelines on evaluation of drug efficacy against protozoa in companion animals [20], without any harmful invasive procedures to the animals. This project was evaluated and approved by the Ethics Committee of VetAgro Sup (approval number 1927).

### Dogs and housing

**Figure 1:**
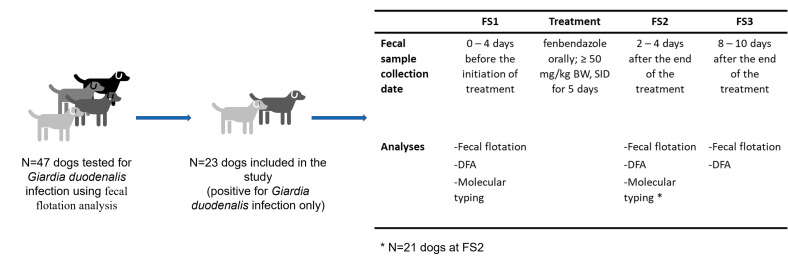
Workflow of the study protocol. FS: fecal samples, DFA: direct immunofluorescence assay

The study took place between April 1, 2019, and March 15, 2020. During this period, any veterinary student at the VetAgro Sup campus (Marcy l’Etoile, France) whose dog presented with diarrhea in the last week but with a good general condition was asked to consent to a fecal flotation analysis (see below) to screen their dog for *Giardia* infection (Fig. 1). Asymptomatic dogs with normal fecal consistency but living in the same household as a *Giardia*-infected dog were also screened for giardiosis.

Among these dogs, only dogs with positive fecal flotation for *G. duodenalis* were included in the study. All dogs that had received drug treatment (such as anthelmintic treatment or immunosuppressive treatment) during the 7 days prior to analysis or dogs that underwent surgery or hospitalization less than 14 days prior to analysis were excluded. Females known to be pregnant were also excluded. Dogs coinfected with other parasites detected through fecal examination were likewise excluded.

Data regarding sex, age, breed, weight, lifestyle, clinical signs, date of onset and date of last deworming for each dog were recorded. Dogs were housed individually in apartments at the veterinary college campus and met other dogs daily in the campus yard. The campus is located next to a forest crossed by a river where all dogs could bathe and drink. No other public water bowl was available on campus.

### Medical treatment and hygiene measures

After fecal diagnosis of giardiosis, all dogs included in the study received fenbendazole (Panacur 500 mg^®^, MSD Animal Health, USA) orally at the minimum dose of 50 mg/kg bodyweight once daily for 5 consecutive days. The drug was given according to the directions on the product label (tablets diluted in water and poured on dog food).

In order to reduce the number of *Giardia* cysts and prevent recontamination, owners were asked to bathe their dogs on day 3 of treatment, by lathering with a grooming shampoo (not necessarily containing chlorhexidine) and rinsing. We also recommended that dog owners clean their apartment and the dog’s bed and bowls with a quaternary ammonium solution or sodium hypochlorite solution [16, 38].

### Fecal sample (FS) and data collection

For each dog included in the study, fresh fecal samples were collected directly by the owners: (1) one fecal sample was collected 0–4 days before the initiation of treatment (FS1); (2) one fecal sample was collected 2–4 days after the end of treatment (FS2; i.e., before the prepatent period of the parasite, which is 4–16 days) [4], and (3) one fecal sample was collected 8–10 days after the end of treatment (FS3; Fig. 1). Fecal sample FS1 corresponds to the initial diagnostic fecal sample before commencement of the study. For each fecal collection time-point, the owner was asked to determine if the dog had diarrhea, intermittent diarrhea or no diarrhea. Intermittent diarrhea was defined as alternating diarrhea (soft or watery feces) and normally formed feces. No fecal score was used, and clinical assessment relied on the owner.

Each fecal sample (FS1, FS2 and FS3) was directly brought or shipped to the parasitology unit of VetAgro Sup for analysis. Fecal samples were stored at +4 °C for no longer than 4 days before fecal analysis and 1 g of each sample was frozen at −20 °C for future studies.

### Fecal flotation

Microscopic examination of fecal samples was first performed by diluting 5 g of feces in 20 mL of zinc sulphate (ZnSO_4_, specific gravity 1.36) [14]. After homogenization, the suspension was strained through one layer of gauze. A tube was filled with the suspension and covered with a coverslip, then centrifuged for 5 min at 600 rpm. The coverslip was placed on a microscope slide, analyzed, and a *Giardia* cyst score was determined according to the number of cysts per slide (Supplementary Data S2). At the same time, samples were also screened for other parasites such as *Cystoisospora* oocysts and nematode eggs.

### Direct immunofluorescence assay

Fresh fecal samples were also examined using a direct quantitative immunofluorescence assay (DFA). This diagnostic method is highly sensitive and specific and is commonly used as the gold standard method for detecting *G. duodenalis* cysts [9, 44]. A Merifluor^®^ Cryptosporidium/Giardia kit (Meridian Bioscience, Cincinnati, OH, USA) was used according to the manufacturer’s instructions. We first diluted 5 g of feces in 15 mL of NaCl 0.9% before collecting 10 μL samples from this solution for analyses. Positive and negative controls were evaluated each time a fecal sample was tested. We used fluorescein isothiocyanate-labeled monoclonal antibodies directed against *G. duodenalis* cell wall antigens and counted each 8–12 μm oval-shaped cyst stained a bright apple green color. The number of cysts observed in the treated slide well after the test procedure was multiplied by 300 to calculate the number of cysts per gram (cpg) of feces. This assay has a theoretical minimum level of detection of 300 cpg of feces and no maximum level of detection.

### Molecular typing

DNA extraction was performed on each FS1 and FS2 fecal sample. In a cryotube, we mixed 250 μg of each frozen sample with a lysis buffer (ASL buffer, Qiagen GmbH, Hilden, Germany). Then, cryotubes were successively frozen with liquid nitrogen and heated at 95 °C (6 cycles, 2 min per step) in order to break cyst walls. Finally, DNA extraction was performed using a QIAamp DNA Stool mini Kit^®^ (Qiagen GmbH, Hilden, Germany), according to the manufacturer’s instructions. To assess the presence of DNA, total nucleic acid quantification was performed with Take3 Microvolume Plates and a Synergy H1^®^ microplate reader (BioTek, Winooski, VT, USA).

A nested polymerase chain reaction (PCR) was performed to yield a fragment of 175 base pairs of the SSU _r_DNA gene. Primers used for the first-round PCR were RH11, 5^′^-CATCCGGTCGATCCTGCC-3^′^ and RH4, 5^′^-AGTCGAACCCTGATTCTCCGCCAGG-3^′^ from Hopkins et al. [22]. PCR amplification was performed in 25 μL volume, with a final mix containing 5–100 ng DNA, 20% 5X Q-Solution (Qiagen GmbH, Hilden, Germany), 10% 10X PCR Buffer (Qiagen, Germany), 0.4 μm of each primer, 1 unit HotStarTaq Plus DNA polymerase (Qiagen GmbH, Hilden, Germany), 400 μm of each dNTP, 3 mm MgCl_2_ and H_2_O. Reactions were heated to 96 °C for 5 min followed by 35 cycles at 96 °C for 45 s, 55 °C for 30 s and 72 °C for 45 s and final elongation at 72 °C for 7 min. Second-round PCR primers used were GiarF, 5^′^-GACGCTCTCCCCAAGGAC-3^′^ and GiarR, 5^′^-CTGCGTCACGCTGCTCG-3^′^ from Read et al., with the same PCR conditions [35]. PCR products were visualized on an ethidium bromide-stained 1.5% agarose gel with TBE buffer and PCR products were sequenced in both directions using the GiarR/GiarF primers. In order to determine the assemblage of each isolate, sequences were aligned and compared with published sequences from GenBank with Blast.

### Statistical analysis

Quantification results for *Giardia* cysts in fecal samples before (FS1) and after (FS2 and FS3) treatment were compared statistically with a Kruskal–Wallis test (non-normal distribution). The heterogeneity of excretion between dogs was high, and the data did not follow a normal distribution (Supplementary Data S2). Therefore, arithmetic means would not have been relevant. Following the World Association for the Advancement of Veterinary Parasitology (WAAVP) guideline recommendations, we used geometrical mean parasite counts to calculate mean cyst excretion before and after treatment.

The percent reduction in cyst shedding was calculated using geometric means as follows:

$$\%\;\mathrm{reduction}=100\;\times \;\left[1-\;\frac{{\mathrm{Mean}}_{\mathrm{after}\;\mathrm{treatment}}}{{\mathrm{Mean}}_{\mathrm{before}\;\mathrm{treatment}}}\right].$$

The theoretical detection limit of the DFA examination was 300 cpg, so any negative DFA examination was recorded as a parasite count of 150 cpg (half of the detection limit of the diagnostic assay, as recommended by the WAAVP [20]).

Dogs were classified into 3 different groups according to the reduction of *Giardia* cysts in fecal samples by DFA examination between FS1 and FS2 only: (1) the G1 group included dogs with ≥90% reduction (minimum efficacy required for efficacy approval according to the WAAVP [20]); (2) the G2 group included dogs with ≥50% and <90% reduction (partial effectiveness), and (3) the G3 group included dogs with <50% reduction (little or no impact of treatment on parasite excretion).

Levels of cyst shedding were compared among groups using a Wilcoxon signed-rank test (non-normal distribution). Reduction of clinical signs after treatment (i.e., from persistent diarrhea to intermittent diarrhea or absence of diarrhea) was also interpreted through a McNemar’s test.

All analyses were performed with R v.4.0.3 software [33].

## Results

### Recruitment of dogs and giardiosis before fenbendazole treatment (FS1)

A total of 47 dogs were recruited for a fecal flotation analysis (Fig. 1). Among these dogs, 24 were infected with other digestive parasites (e.g., *Cystoisospora* sp., *Ancylostoma* sp., *Toxocara* sp.), with or without concomitant infection with *G. duodenalis*. As co-infection could interfere with the results of the trial, these dogs were excluded from the study.

**Figure 2:**
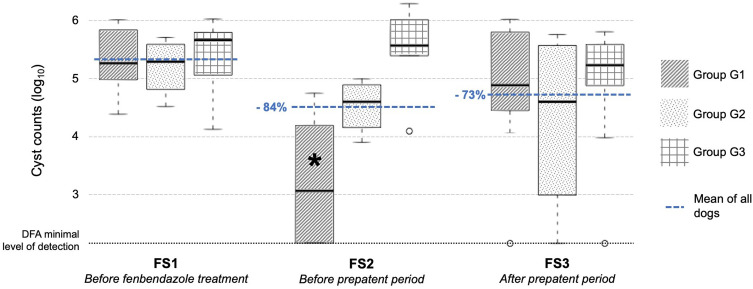
Cyst shedding for G1, G2 and G3 groups at FS1, FS2 and FS3 (cyst counts in log_10_). FS1: fecal samples collected 0–4 days before initiation of treatment, FS2: fecal samples collected 2–4 days after the end of treatment, FS3: fecal samples collected 8–10 days after the end of treatment, G1: dogs with reduction of cysts ≥ 90% between FS1 and FS2, G2: dogs with reduction of cysts ≥ 50% and <90% between FS1 and FS2, G3: dogs with reduction of cysts < 50% between FS1 and FS2, DFA = direct immunofluorescence assay, *: significant reduction in cyst shedding compared with FS1.

The remaining 23 dogs (11 males and 12 females) were positive for *G. duodenalis* only and were included in the study (Table 1). Breeds were heterogeneous and dogs had an average age of 8 ± 3.8 months and an average weight of 15.7 ± 2.8 kg (Supplementary Data S1). Before treatment, the geometric mean excretion of *Giardia* cysts, evaluated by DFA analysis, was 213,989 cysts per gram (cpg) of feces (interquartile range (IQR) = 97,500–555,600 cpg) (Fig. 2). Then, dogs received fenbendazole orally at an average dose of 58 ± 2 mg/kg for 5 consecutive days. All owners reported having shampooed their dogs and cleaned their house, as prescribed. There was no significant difference in cyst excretion between G1, G2 and G3 groups at FS1 (*p* > 0.05, Kruskal–Wallis test).

**Table 1:** Study design and fecal examination methods for all the time-points of fecal collection in 23 dogs.

	FS1	Treatment	FS2	FS3
Day of collection	0–4 days before the initiation of treatment	Fenbendazole 50 mg/kg BW once daily for 5 consecutive days + disinfection of dog and environment	2–4 days after the end of treatment	8–10 days after the end of treatment
Fecal consistency (number of dogs with diarrhea/intermittent/no diarrhea)	21/0/2		5/11/7	9/4/10
Fecal flotation (positive/negative results)	23/0		19/4	17/5
Direct immunofluorescence assay (geometrical mean)	213,989 cpg (IQR = 97,500–555,600)		34,147 cpg (IQR = 9675–333,600)	57,239 cpg (IQR = 12,000–456,000)
Molecular typing by PCR (C/D/C + D)	15/4/0		14/5/1	Not evaluated

BW: body weight, cpg: cysts per gram, IQR: interquartile range, PCR: polymerase chain reaction.

### Elimination of *Giardia* cysts after treatment and before the prepatent period (FS2)

After treatment but before the prepatent period of the parasite (FS2; prepatent period of the parasite: 4–16 days) [4], the geometric mean fecal excretion of *Giardia* cysts was 34,147 cpg (IQR = 9675 – 333,600) according to DFA quantification (Fig. 2). Excretion dropped by 84% (95% Confidence Interval (CI) = 83.9%–84.2%) between FS1 and FS2, although this was not significant (*p* = 0.198, Wilcoxon signed-rank test). However, heterogeneous trends among dogs were observed, with high (G1; *n* = 8), partial (G2; *n* = 4) or minimal (G3; *n* = 11) reduction of *Giardia* cyst shedding. Significant cyst reduction was observed in G1 (*p* = 0.008) but not in G2 (*p* = 0.125) or G3 (*p* = 0.193). No *Giardia* cysts were detected by DFA and fecal flotation examination in 4 dogs, all from the G1 group. Geometric mean excretion of *Giardia* cysts after treatment (FS2) in G1, G2 and G3 were 1690 cpg (IQR = 150 – 13,875), 33,882 cpg (IQR = 21,600 – 71,775), and 379,359 cpg (IQR = 258,225 – 1,002,600), respectively, with a significant difference in excretion among groups (*p* < 0.05, Kruskal–Wallis test). These data correspond to a reduction immediately after treatment of 99.2% and 79.0% in G1 and G2, respectively, and an increase of 58.4% in G3.

### Elimination of *Giardia* cysts after treatment and after the prepatent period (FS3)

After treatment and after the prepatent period (FS3), the geometric mean fecal excretion of *Giardia* cysts was 57,239 cpg (IQR = 12,000 – 456,000) (Fig. 2). It dropped between FS1 and FS3 by 73.3% (95% CI = 73.1%−73.4%); however, there was no statistically significant difference compared with excretion before treatment (*p* = 0.272, Wilcoxon signed-rank test). No significant cyst reduction was observed in G1 (*p* = 0.813), G2 (*p* = 0.875) or G3 (*p* = 0.131). No significant difference in excretion between each group was observed at FS3 (*p* > 0.05, Kruskal–Wallis test). Three out of the 4 dogs with negative DFA examination after treatment (FS2) excreted *Giardia* cysts again at FS3. Conversely, 3 dogs in G3 had a 90% reduction in *Giardia* cyst excretion at FS3 even if they did not have any cyst reduction immediately after treatment.

Dogs in G1 excreted more cysts at FS3 compared with FS2, with a geometric mean excretion of 64,353 cpg (IQR = 41,250 – 688,200), whereas dogs in G2 and G3 excreted fewer cysts (geometric mean excretion of 19,720 cpg, IQR = 5212–333,000 and 80,759 cpg, IQR = 78,525–372,300, respectively).

No *Giardia* cysts were detected by DFA in 3 dogs at FS3 (1 dog in each group).

### Fecal consistency

Before fenbendazole treatment, 21 dogs presented with diarrhea and 2 dogs were asymptomatic. These 2 adult dogs (1 and 3 years old), who were included because they lived in the same household as a *Giardia*-infected dog, remained asymptomatic during the study period. Only 5 of the symptomatic dogs continued to suffer from persistent diarrhea after treatment (all belonging to G3). The other dogs had intermittent (G1 = 4/7; G2 = 3/4; G3 = 4/10) or no (G1 = 3/7; G2 = 1/4; G3 = 1/10) diarrhea. These data suggest that fenbendazole treatment can reduce clinical signs in 76.2% of dogs initially presenting for diarrhea (*p* < 0.001, McNemar’s test). At FS3, only 8 dogs showed persistent diarrhea (G1 = 0/7; G2 = 1/4; G3 = 7/10), with a reduction of clinical signs in 61.9% of dogs (*p* = 0.003, McNemar’s test).

### Assemblages

Molecular typing was performed before and after treatment (FS1 and FS2) on 21 dogs: 2 dogs were excluded because of lack of fecal material (1 dog in G1 and 1 dog in G3). Positive PCR results at the SSU _r_DNA locus were obtained in 92.9% (39/42) of submitted samples. PCR failed to amplify 3 isolates despite detection of cysts during DFA examination (FS1 and FS2 samples of dog No. 15 in G2 and FS1 sample of dog No. 18 in G3). Conversely, all isolates (4/4) from samples that were negative on both fecal flotation examination and DFA examination were successfully amplified with PCR. Finally, we successfully performed DNA sequencing of a 175 bp SSU _r_DNA gene in all PCR-positive dogs (39/39).

**Figure 3:**
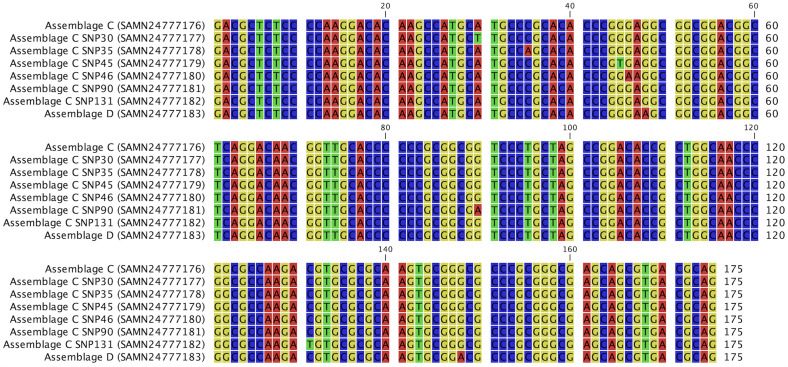
Sequences of assemblage C, assemblage C with nucleotide substitutions and assemblage D obtained at the SSU _r_DNA gene locus. GenBank references are written in brackets. SNP: single nucleotide polymorphism.

Dogs harbored assemblage C in 76.9% of samples (30/39) and assemblage D in 25.6% of samples (10/39). In 1 sample, coinfection with both assemblages C and D was observed (proportion not known). No zoonotic assemblage (A or B) was found. All sequences were uploaded as GenBank numbers (reference PRJNA795798). Among samples with the assemblage C nucleotide sequence (30/39), 23.3% (5 dogs at FS1 and 2 dogs at FS2) exhibited single nucleotide substitutions that were not previously reported in GenBank references (similarity of 99.4%; Fig. 3). Similarity with sequences for assemblage D was 100%.

Before fenbendazole treatment (FS1), 78.9% of dogs (15/19) shed *Giardia* cysts from assemblage C and 21.1% (4/19) from assemblage D. After fenbendazole treatment (FS2), 75% of dogs (15/20) excreted *Giardia* cysts from assemblage C and 30% (6/20) from assemblage D, including one dog that excreted both assemblages C and D. After excluding dogs with negative zinc sulfate centrifugation and DFA results (*n* = 4), 81% of remaining dogs (13/16) excreted *Giardia* cysts from assemblage C and 25% (4/16) from assemblage D at FS2. Therefore, the assemblage detected at FS2 (after fenbendazole treatment) was different to the assemblage detected at FS1 in 36.8% (7/19) of the dogs. Excluding dogs with negative zinc sulfate centrifugation and DFA results (*n* = 4), assemblage changes occurred in 33.3% (5/15) of dogs. The observed changes were either from assemblage C to D (4/15) or from assemblage D to C (3/4). Changes were reported equally in G1 (3/7), G2 (2/7) and G3 (2/7) and did not seem to be associated with treatment efficacy. Assemblages did not match with a specific group and were equally distributed among groups before and after treatment. Likewise, samples with nucleotide substitutions were reported in the same proportion among groups (G1 = 3/7; G2 = 1/7; G3 = 3/7) and did not appear to influence treatment efficacy.

## Discussion

Five consecutive days of treatment with fenbendazole 50 mg/kg orally once daily led to a mean reduction in *Giardia* cyst shedding of 84% 2–4 days after the end of treatment (FS2) and a reduction of 73% 8–10 days after the end of treatment (FS3). A reduction of 90% or more was not reached so therapeutic efficacy cannot be claimed according to the WAAVP guidelines on evaluation of drug efficacy against gastrointestinal protozoa in companion animals [20]. In addition, cyst shedding was highly variable among dogs and only 17% of dogs (4/23) showed complete elimination of *G. duodenalis* after treatment. In parallel, dog owners observed a reduction in clinical signs in the majority of dogs after treatment (76% (16/21) and 62% (13/21) of dogs at FS2 and FS3, respectively). All dogs with a reduction in *Giardia* cyst excretion (G1 and G2) also had improved fecal consistency, whereas only half of the dogs (5/10) in G3 had improved fecal consistency.

Between FS1 and FS2, only 35% of dogs (G1, 8/23) achieved therapeutic success (≥90% reduction of cysts), presumably due to fenbendazole treatment. However, the rate of therapeutic success may be lower as we cannot rule out spontaneous recovery in some individuals due to the absence of a control group. In fact, without treatment, giardiosis in dogs is self-limiting and cyst shedding stops after 27–35 days of infection in most dogs, although some dogs can remain infected for several months [24]. Trophozoite elimination in untreated dogs is the consequence of humoral immunity with an elevated anti-*Giardia* IgG or IgA response [31]. Therefore, the observed reduction in *Giardia* cysts may partly be a consequence of an effective immune response during the study period.

Despite treatment, almost half of the dogs (G3, 11/23) continued to release *Giardia* cysts at a high level after the end of treatment (FS2). At that time (2–4 days after the end of fenbendazole treatment), recontamination should not occur as analyses were performed before the prepatent period of the parasite (4–16 days) [4]. Therefore, therapeutic failure or chemoresistance could be suspected in G3 dogs. Treatment was administered by veterinary students, so medication compliance was assumed to be correct. However, although extensive instructions were given to these well-informed owners, and they all confirmed adherence to these instructions, there may have been differences in the quantity of drug ingested by dogs with their food when following the product label instructions (tablets diluted in water and poured on dog feed). Administration of tablets directly into dogs’ mouths may have reduced the potential risk of uncertainty in drug ingestion, but this is not recommended as it can reduce absorption and bioavailability [34]. Reappearance of *Giardia* cysts at FS3 could be due to recontamination through the environment after the end of treatment. Sources of reinfection remained present during the study with shared river and facilities on campus. This, associated with stress and non-standardized diet and water consumption, could lead to reinfection after treatment, with positive results 8–10 days after treatment (FS3). Therefore, despite strict hygiene measures, reinfections and/or multiplication of *Giardia* cysts could explain a higher cyst excretion level at FS3 compared with FS2 in G1 dogs. Cases of reinfection have been reported in other home condition field trials similar to our study [11].

The Companion Animal Parasite Council (CAPC) [10] and European Scientific Counsel for Companion Animal Parasites (ESCCAP) [16] guidelines on the control of *G. duodenalis* in companion animals promote the efficacy of fenbendazole with the recommended dosage of 50 mg/kg orally once daily for 5 consecutive days. Among the few studies assessing the efficacy of fenbendazole against giardiosis, only the latest [11] used the recommended dosage and duration. In that study, 67% of dogs (8/12) tested negative for *Giardia* cysts after treatment (compared with a negative rate of 17% (4/23) and an 84% reduction in *Giardia* cyst shedding in our study). However, the study used only fecal flotation examination and therefore cannot be compared with our study because we used DFA examination to quantify cyst shedding. With fenbendazole at a dose of 50 mg/kg once for only 3 consecutive days, the negative *Giardia* rate after treatment varies from 0% [12] to 100% [47] and reduction in *Giardia* cyst shedding varies from 30% [18] to 84% [27]. The treatment-refractory cases observed in our study, especially in the G2 group, where cyst reduction was between 50% and 90%, could be explained by a lack of treatment observance or chemoresistance. In these dogs, most cysts were eliminated by the treatment, whereas a minority of cysts survived.

Chemoresistance, or drug resistance, is defined by the World Health Organization as “the ability of a parasite strain to survive and/or multiply despite the administration and absorption of a drug in doses equal to or higher than those usually recommended but within the limits of tolerance of the subject” [25]. Chemoresistance must be differentiated from recontamination or treatment failure due to a lack of treatment observance. *Giardia duodenalis* has been facing high drug pressure with fenbendazole for several years on the present study site, potentially leading to the selection of chemoresistant strains. Further studies should be performed to investigate the different factors, such as treatment administration and chemoresistance, that might explain the persistence of *G. duodenalis* cysts despite treatment at the recommended dosage. In dogs, benzimidazole chemoresistance is well known with *Ancylostoma caninum* [15, 23]; however, chemoresistance with *G. duodenalis* has only been studied *in vitro* [1]. Biochemical studies of isolates from benzimidazole-refractory giardiosis cases (human or animal) have not been reported to date [1]. Furthermore, culturing *Giardia* strains is challenging and may alter the initial composition and genetic diversity present in the infected host [41]. Resistance markers are not available in daily practice to assess the presence of chemoresistance. Therefore, little is known about benzimidazole resistance in *Giardia* trophozoites [1]. *In vitro* chemoresistance could be due to different mechanisms, including dysregulation and epigenetic changes of genes other than the β-tubulin gene, such as those encoding α-2-giardin, β-giardin, ran binding protein 1 or antioxidant enzymes [1]. Currently, most of these results come from axenic culture derived from WB strains (assemblage A), and not from canine-specific assemblages, so transposition could be partially biased. Animals may shed *Giardia* cysts intermittently and, therefore, some authors recommend 2 or 3 successive fecal flotation examinations to detect giardiosis [13]. These variable results are mainly due to the low sensitivity of zinc sulphate centrifugation. Bayesian studies showed that the sensitivity of zinc sulphate centrifugation could be as low as 26.4–48.2% [42, 45], even when the examination was performed by well-trained technicians. However, the direct immunofluorescence assay used in our study had high sensitivity and specificity even on one fecal sample. Similarly, Bayesian analysis conducted on DFA data generated by the Merifluor^®^ kit found sensitivity of 91% in symptomatic dogs [19] and 78.6% in symptomatic and asymptomatic dogs [45], as well as high specificity (94–97%). According to the sensitivity of these tests, false negative results due to misdiagnosis of *Giardia* cyst absence on both DFA and direct zinc sulphate centrifugation examination are improbable but still possible. Therefore, collecting and analyzing 2–3 fecal samples over time may limit the influence of temporal variation in the shedding of *Giardia* cysts [13]. PCR and coproantigen tests are not good follow-up diagnostic tests for this type of study, because they remain positive for at least 1 week and several weeks, respectively, after complete elimination of the parasite [7, 36]. This could be due to detection of cyst antigen or *G. duodenalis* DNA that may remain in the feces, even after the parasite has been destroyed [7]. Therefore, these tests do not assess the viability of *Giardia* cysts after treatment, whereas DFA examination can differentiate viable from non-viable cysts. Interestingly, isolates were successfully amplified by PCR from all 4 fecal samples with both a negative fecal flotation examination and DFA examination result at FS2 (i.e., 2–4 days after the end of treatment).

We performed *Giardia* molecular typing using a nested PCR [22, 35] and sequencing of a SSU _r_DNA 175 bp locus. In agreement with previous studies in which assemblages C and D were largely predominant in dogs [6, 39], in our study, assemblage C was present in 76.9% of samples (30/39) and assemblage D in 25.6% of samples (10/39). Only 1 dog in our study had cysts of assemblages C and D. Moreover, we did not detect shedding of zoonotic assemblages (A or B) in any of the dogs in our study. Zoonotic assemblages are considered to be more prevalent in dogs living alone with their owner [26], while dogs recruited in our study were all living in the same campus yard and were frequently in contact with other dogs. Neither assemblage C nor assemblage D were overrepresented in 1 particular group and treatment efficacy was not linked to the initial infecting assemblage. However, intra-assemblage subspecies of *G. duodenalis* were not reported in our study due to limits of use of the SSU _r_DNA locus. Use of only 1 targeted locus can lead to underestimation of the diversity of genotypic assemblages as assemblage swapping can occur. This can be detected by multilocus sequence typing.

Despite the conserved nature of the SSU _r_DNA locus, in this study we report 6 different new nucleotide substitutions in assemblage C sequences, compared with the consensus sequence, that have not been reported previously in GenBank (Fig. 3). These nucleotide substitutions were equally distributed among the groups. They were found mostly before treatment (in 6/7 dogs) and not after treatment, at which point they were replaced by standard assemblage sequences.

Interestingly, 36.8% of dogs presented a change of assemblage between FS1 and FS2 (i.e., before and after the end of treatment). No recontamination can occur at that time, due to the prepatent period of the parasite exceeding 4 days. These changes of assemblage could be explained by an unequal efficacy of fenbendazole treatment on selected subpopulations of *G. duodenalis*. We can assume that several subpopulations of *G. duodenalis* may have coexisted before treatment and that these subpopulations were not detected because our PCR may only have detected the predominant assemblage. Treatment may significantly reduce some subpopulations of *G. duodenalis* and, therefore, the presence of minor subpopulations may be more easily detected after treatment. In addition, the assemblages did not seem to be associated with lower treatment efficacy, as assemblage C and D were not different in G1 dogs before and after treatment. Use of multilocus sequence typing, with the ITS1-5.8S-ITS2, glutamate dehydrogenase, triosephosphate isomerase or beta-giardin locus, is needed to confirm our findings [6, 32].

The current study was a field trial in home conditions in dogs naturally infected by *Giardia* and was within normal conditions of use in the field. Our study had a relatively large cohort of dogs (23 dogs) compared with other studies [11, 12, 29]. Major limitations are the absence of a placebo or control group for ethical reasons and the absence of a comparable group (such as a metronidazole-treated group). This means that all study criteria from the WAAVP guidelines for evaluating the efficacy of drugs against non-coccidial gastrointestinal protozoa were not met. General recommendations for study design in home conditions were followed, but dogs were not parsed into a control group as recommended. Despite these limitations, our study highlights the unsatisfactory efficacy of fenbendazole against giardiosis, with only 35% of dogs achieving therapeutic success (reduction of cysts ≥ 90%) and only 17% of dogs experiencing complete elimination of parasites. However, our data suggest that fenbendazole may help in the management of giardiosis, as shedding of *Giardia* cysts dropped significantly by 84% 2–4 days after the end of treatment. Similarly, clinical signs decreased in 76% of dogs after the end of treatment. Hygiene measures and disinfection of the environment appeared to be essential to avoid recontamination. Further studies with a placebo group and larger group sizes are still needed to confirm our results, and to assess the efficacy of fenbendazole for the treatment of giardiosis in home conditions.

## Conflict of interest

The authors declare that they have no conflicts of interest.

## Supplementary materials

Supplementary material is available at https://www.parasite-journal.org/10.1051/parasite/2022048/olm.
Supplementary Table S1:
Characteristics, duration of clinical signs and deworming habit of dogs included in the study.
Supplementary Table S2:
Results of the zinc sulfate centrifugation (ZSC), direct immunofluorescent assay (DFA), genotyping (PCR) and the fecal consistency (FC) of each isolate 0–4 days before the initiation of treatment (FS1), 2–4 days after the end of treatment (FS2) and 8–10 days after the end of treatment (FS3).
