# Supplementary material for: Lack of efficacy of fenbendazole against Giardia duodenalis in a naturally infected population of dogs in France
Source: Parasite. 2022 Oct 28;29:49. doi: 10.1051/parasite/2022048 (PMC9621113; doi:10.1051/parasite/2022048)
Supplement: Supplementary Table S1: — Characteristics, duration of clinical signs and deworming habit of dogs included in the study. [file parasite-29-49-s1.pdf]

Lack of efficacy of fenbendazole against *Giardia duodenalis* in a naturally infected population of dogs in France

Supplementary data

Hugo Kaufmann[1], Lionel Zenner[1,2], Slimania Benabed[1,2], Marie-Thérèse Poirel[1,2], Gilles Bourgoin[1,2]

[1] Université de Lyon, VetAgro Sup - Campus Vétérinaire de Lyon, Laboratoire de parasitologie vétérinaire, 1 avenue Bourgelat, BP 83, F-69280 Marcy l'Etoile, France.

[2] Université de Lyon, Université Lyon 1, CNRS, UMR 5558, Laboratoire de Biométrie et Biologie Évolutive F-69622 Villeurbanne, France.

*Corresponding author: Gilles Bourgoin[1,2], gilles.bourgoin@vetagro-sup.fr

Table S1. Characteristics, duration of clinical signs and deworming habit of dogs included in the study

Dog no.	Age (months)	Sex	Weight (kg)	Breed	Duration of clinical signs (days)	Previous exposure to bdz
01	5	Male	9	Border Collie	14	Yes
02	4.5	Female	11	Dalmatian	10	Yes
03	4.5	Male	13.5	Australian Shepherd	> 21	Yes
04	6	Female	25	Beauceron	> 21	Yes
05	9.5	Female	29.5	Beauceron	> 21	No
06	15	Female	29	White Swiss Shepherd	> 21	Yes
07	4	Male	15	White Swiss Shepherd	> 21	Yes
08	22.5	Female	17	Mixed breed	> 21	No
09	4	Male	17	Leonberger	1	No
10	2.5	Male	11	Beauceron	3	No
11	2.5	Female	5	Mixed breed	3	No
12	41	Male	21.5	Australian Cattle	Asymptomatic	-
13	13	Female	18	Mixed breed	2	No
14	4	Female	12	Mixed breed	14	No
15	2.5	Male	20.2	Autralian Shepherd	> 21	Yes

16	4	Female	8	Autralian Shepherd	> 21	Yes
17	4	Female	9.1	Mixed breed	> 21	Yes
18	4	Male	9.9	Finnish Lapphund	2	Yes
19	5	Male	12.5	Mixed breed	> 21	No
20	3.5	Male	14.2	Golden Retriever	> 21	Yes
21	12	Female	20.5	Autralian Shepherd	Asymptomatic	Yes
22	5.5	Male	17	Mixed breed	3	Yes
23	5.5	Female	15.1	Mixed breed	> 21	Yes
