# Supplementary material for: Lack of efficacy of fenbendazole against Giardia duodenalis in a naturally infected population of dogs in France
Source: Parasite. 2022 Oct 28;29:49. doi: 10.1051/parasite/2022048 (PMC9621113; doi:10.1051/parasite/2022048)
Supplement: Supplementary Table S2: — Results of the zinc sulfate centrifugation (ZSC), direct immunofluorescent assay (DFA), genotyping (PCR) and the fecal consistency (FC) of each isolate 0–4 days before the initiation of treatment (FS1), 2–4 days after the end of treatment (FS2) and 8–10 days after the end of treatment (FS3). [file parasite-29-49-s2.pdf]

Table S2. Results of the zinc sulfate centrifugation (ZSC), direct immunofluorescent assay (DFA), genotyping (PCR) and the fecal consistency (FC) of each isolate 0–4 days before the initiation of treatment (FS1), 2–4 days after the end of treatment (FS2) and 8–10 days after the end of treatment (FS3).

Dog	FS1				FS2				FS3		
no.	ZSC	DFA	PCR	FC	ZSC	DFA	PCR	FC	ZSC	DFA	FC
01	2	265 200	C	2	3*	251 100	C	2	1	9 900	0
02	2	13 500	C	2	5	12 600	C	2	-	-	2
03	5	986 400	C	2	4	1 093200	D	2	2	79 200	2
04	4	487 200	C	2	4	279 600	C	2	3	78 300	2
05	4	24 600	C	2	0	Neg	D	0	1	12 000	0
06	3	33 300	C	2	0	8 100	C	1	0	Neg	0
07	3	810 000	D	2	2	56 700	C	0	1	-	0
08	2	82 500	C	2	0	Neg	C	1	0	Neg	0
09	4	465 600	D	2	3	250 800	D	1	1	290 400	2
10	2	31 800	C	2	5	351 600	C	1	2	661 200	2
11	4	141 300	M	2	0*	Neg	C	1	0*	79 200	1
12	2	61 800	-	0	3	-	-	0	2	152 400	0
13	4	113 100	M	2	0	Neg	D	0	1	70 500	0
14	4	244 800	D	2	2*	9 300	C	1	2	456 000	0
15	2	516 000	Neg	2	2	62 400	Neg	0	2	246 000	1
16	4	512 400	C	2	5*	871 200	C	1	3	201 600	2
17	3	298 800	D	2	3	99 900	M	1	0	6 900	2
18	3	782 400	Neg	2	3	1 046 400	M	0	4	543 600	0
19	2	219 600	M	2	4	400 800	D	1	2	399 600	1
20	5	1 072 800	M	2	5	1 981 200	C	2	0*	Neg	2
21	2	1 036 800	-	0	2	10 800	-	0	4	1 083 600	0
22	5	595 200	M	2	1	23 100	P	1	1	920 400	1
23	2	131 100	C	2	2	26 100	C	1	1	594 000	0

ZSC: 0 = absence of cyst on the sample, 1 = 1-10 cysts per slide, 2 = 10–100 cysts per slide, 3 = 100–200 cysts per slide, 4 = 200–1000 cysts per slide, 5 = more than 1000 cysts per slide, * = Co-infection with *Cystoisospora* oocysts. SFA: Cysts per gram of feces (cpg). A parasite count of 150 cpg was associated to negative result (Neg).

PCR: Assemblage using the SSU $_r$DNA locus. C = assemblage C, D = assemblage D, M = assemblage C with single nucleotide substitutions, P = coinfection with assemblage C and D. FC: 0 = Absence of diarrhea, 1 = Intermittent diarrhea, 2 = Diarrhea.
